# Hybrid Silicon Substrate FinFET-Metal Insulator Metal (MIM) Memristor Based Sense Amplifier Design for the Non-Volatile SRAM Cell

**DOI:** 10.3390/mi14020232

**Published:** 2023-01-17

**Authors:** G. Lakshmi Priya, Namita Rawat, Abhishek Sanagavarapu, M. Venkatesh, A. Andrew Roobert

**Affiliations:** 1 Centre for Innovation and Product Development, Vellore Institute of Technology, Chennai 600127, India; 2 School of Electronics Engineering, Vellore Institute of Technology, Chennai 600127, India; 3 Department of ECE, CMR Institute of Technology, Bengaluru 560037, India; 4 Department of ECE, Francis Xavier Engineering College, Tirunelveli 627003, India

**Keywords:** silicon substrate FinFET, MIM Memristor, sense amplifier, low power SRAM, Monte-Carlo simulation

## Abstract

Maintaining power consumption has become a critical hurdle in the manufacturing process as CMOS technologies continue to be downscaled. The longevity of portable gadgets is reduced as power usage increases. As a result, less-cost, high-density, less-power, and better-performance memory devices are in great demand in the electronics industry for a wide range of applications, including Internet of Things (IoT) and electronic devices like laptops and smartphones. All of the specifications for designing a non-volatile memory will benefit from the use of memristors. In addition to being non-volatile, memristive devices are also characterized by the high switching frequency, low wattage requirement, and compact size. Traditional transistors can be replaced by silicon substrate-based FinFETs, which are substantially more efficient in terms of area and power, to improve the design. As a result, the design of non-volatile SRAM cell in conjunction with silicon substrate-based FinFET and Metal Insulator Metal (MIM) based Memristor is proposed and compared to traditional SRAMs. The power consumption of the proposed hybrid design has outperformed the standard Silicon substrate FinFET design by 91.8% better. It has also been reported that the delay for the suggested design is actually quite a bit shorter, coming in at approximately 1.989 ps. The proposed architecture has been made significantly more practical for use as a low-power and high-speed memory system because of the incorporation of high-K insulation at the interface of metal regions. In addition, Monte Carlo (MC) simulations have been run for the reported 6T-SRAM designs in order to have a better understanding of the device stability.

## 1. Introduction

SRAMs with low power consumption are becoming increasingly common in VLSI chips. This is particularly true in microprocessors, where on-chip cache capacities are increasing with each iteration to close the growing gap between processor and main memory rates. As integration and running rates have increased, power dissipation has become a critical issue. In recent years, low-wattage IC design has gained attention due to the proliferation of rechargeable devices. The most common method for lowering system power consumption in memory design is to reduce transistor size. Manufacturers of Field Effect Transistor (FET) based scaled CMOS devices are doing everything they can to help achieve this decrease [1,2]. When the approach progresses beyond the 90 nm node and into the sub-nanometer realm, leakage becomes a major worry. When MOS devices hit their scaling limits, the semiconductor industry developed the FinFET, which is now the most popular choice for next generation devices. Moore’s law is being supported by several semiconductor firms investing in FinFET technology. FinFETs feature a completely depleted silicon film (body) that is either undoped or mildly doped.

Due to its speedier operation and higher performance, SRAM is employed as a memory element. In SRAM, however, data preservation is critical since data is destroyed when the power is turned off. Due to its instability, systems that use conventional SRAM like primary memory or cache have quite a longer startup time. Nonvolatile memory can assist minimize startup time and energy consumption [3,4]. It is possible that a CMOS Memristor-based SRAM cell would have been an efficient circuit component that would have allowed standard memory cells to sustain data despite the power being disconnected.

The proposed research will emphasize the developing novel SRAM architecture that tends to make use of FinFET and Memristor technologies. Further, Monte Carlo simulation is done for the proposed model of the hybrid FinFET-Memristor model. This paper is structured as follows: Section 2 presents the implementation design of SRAM design using CMOS, FinFET and Memristor. In Section 3, novel Silicon Substrate FinFET–Ti/TiN Memristor hybrid 6T-SRAM design is explored. Section 4 presents the simulation findings while Section 5 concludes the study.

## 2. Implementation of 6T-SRAM Using CMOS, FinFET & Memristor

### 2.1. Silicon Substrate FinFET

**Figure 1:**
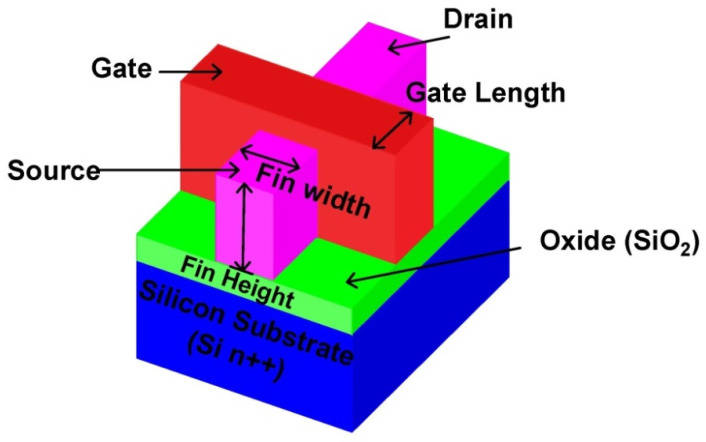
Three-dimensional view of Silicon substrate based FinFET.

FinFET was invented by a research team at California State University, Berkeley, to characterize a quasi, dual-gated transistor constructed on an SOI substrate [5,6,7]. The FinFET technology was born because of the ever-increasing degrees of integration. It is based on Moore’s law, which stipulates that every two years the count of transistors on a particular region of silicon becomes twice. FinFET’s appeal stems from its lower leakage current, higher performance, and a variety of implementation techniques including the usage of Tunnel FETs and Junctionless Tunnel FETs were also used in the literature for the SRAM design [8,9,10,11]. Scaling down, according to Moore’s law, provides a high integration density on the chip and aids in the management of the short channel effect. FinFET’s low power consumption enables high integration levels because of their lower threshold voltage, FinFETs run at a lower voltage. Lower threshold results in their operation at a 30 percent quicker rate than non-FinFET devices. The FinFET is differentiated by its conducting channel, which is constructed of silicon called “fin”, which also acts as the particular device’s body. FinFETs are sometimes known as non-planner double gate MOSFETs (DG-MOSFETs) since the front and rear gates are connected. Figure 1 depicts a typical FinFET structure. According to the architecture, the effective channel width (electrical width) W_eff_, of single-fin FinFET must be twice the fin height [12]. Using numerous Fins allows for a wider device width. By raising, height of the fin and hence channel width, the FinFET’s driving current may be increased. Constructing multiple parallel fins provides larger drive current strengths per unit area.

The W_eff_ of FinFET is formulated as [12]:

W_eff_ = N_fin_ (T_fin_ + 2 H_fin_) (1)

where,

N_fin_ = Number of the parallel fins,

T_fin_ = Fin Thickness (width),

H_fin_ = Height of each fin.

The triple gate electrode FinFET on a heavily doped Silicon substrate (Si n^++^) is presented in Figure 1. The gate length (L) has been chosen to be 32 nm, with an Aluminium (Al) gate metal work function of 4.28 eV on all the three sides of fin. Tunneling places severe constraints on the thickness (tox) of the gate insulating material, which can only be 2.5 nm; meanwhile, the threshold shift from quantum confinement restricts the width of the fins to 0.2 nm. The shortest FinFET channel length attainable with these limiting values is in the region of 5 to 30 nm, thanks to several electrically connected gates and thin silicon film layer that assists in reducing short channel effects (SCE).

**Figure 2:**
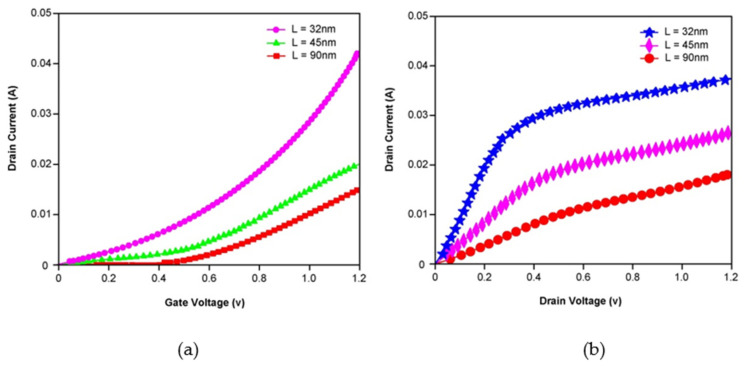
Drain current characteristics of Silicon substrate based FinFET with applied (**a**) Gate Voltage and (**b**) Drain Voltage for various channel lengths of L = 32 nm, 45 nm and 90 nm.

FinFET with gate lengths below 30 nm leads to poor subthreshold behavior of the device. The drain current characteristics of the proposed FinFET are obtained for both the applied gate and drain voltages, as shown in Figure 2. It is well known that the triple gate structure surrounding the fin will cover more channel surface than the traditional planar FETs. This leads to better electrostatic control of the gate over the channel, reduced channel resistance and increased drain current. Figure 2 clearly shows the improvement in drain current for varied channel lengths. With L = 32 nm, the proposed FinFET has achieved a drain current of up to 40 mA with V_GS_ = V_DS_ = 1.2 V.

### 2.2. Novel Metal-Insulator-Metal Based Memristor Model

The fourth passive circuit element is the Memristor since it was conceptualized by Leon Chua in 1971. However, in 2008, D. Structov et al. analyzed memristor considering its physical structure. L. Chau, 1971 [13] led to the discovery of memristors but D. Structov et al., 2008 analyzed memristors considering their physical existence, they have explained how some nano-scales possess properties similar to memristors.

The mathematical models are utilized to recreate device behaviour, which was first achieved by HP laboratories employing TiO_2_-based nanofilms for non-volatile nature. Even the device is not yet available commercially, it is a potential element for low-area, less-power applications, and research is being conducted using various mathematical models. The hysteresis I-V curve detected in Titanium dioxide-based devices seems to be useful for making memristors.

The coupling that occurs between the positively and negatively charged ions in a memristor causes the device to take on the features that are characteristic of a bipolar device. When a voltage bias is given to this device, positively charged oxygen TiO_2_-x vacancies begin to migrate towards one end, therefore lowering the effective resistance of the memristor. When the power is removed, the resistance does not alter. By applying a reverse bias voltage, vacancies can be returned to their source. As a consequence of this, the memristance can be modified between a value of zero (R_on_) and a value of one (R_off_). When the voltage is lower than a threshold (V_th_), this indicates that the memristor is exhibiting its memory feature [14,15].

**Figure 3:**
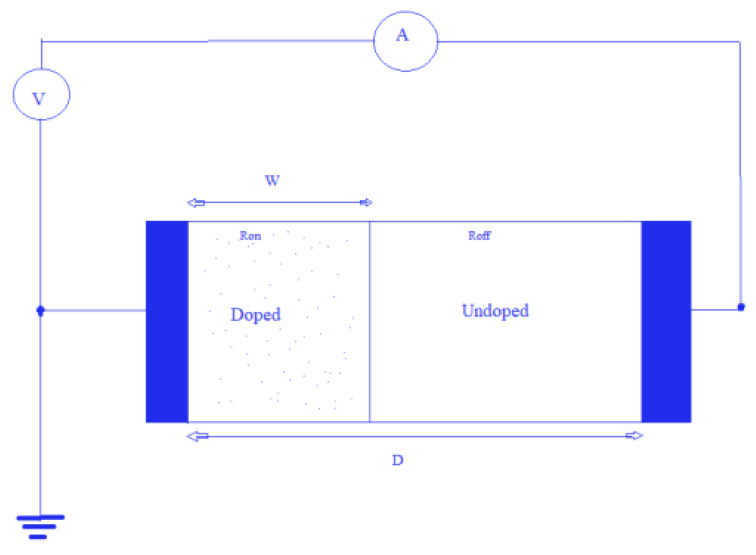
Basic model for Memristor [13].

The linear modeled structure of memristor consisting of two thin-film layers of TiO_2_, by HP is expressed in mathematical terms and also shown in Figure 3. The summation of the resistance of undoped and the oxide doped layers is considered as the total resistance of memristor R_mem_. 

The higher and lower boundaries of the resistance values for W = 0 and W = D are denoted by notations R_on_ and R_off_, respectively [15].

R_mem_ = R_off_(1 − x) + R_on_(x) (2)

The voltage between the two terminals is given by [15]:

V (t) = (R_off_ [1 − W (t)/D] + R_on_ [W (t)/D]) × i (t) (3)

where x = W/D ∈ (0, 1), 

“x” signifies the width of the oxide-doped area in the semiconductor,

D is the aggregated length of regions that are doped and undoped.

When applied to real-world cases, a linear ion drift model is completely ineffective. Some flaws with this paradigm need to be fixed. For instance, the memristor’s internal electric field (MV/cm) has high variability that is disregarded by the linear model. According to the non-linear ion drift model, the voltage-controlled memristor is intended to have a non–linear relationship between potential and the internal state. [16,17]. 

**Figure 4:**
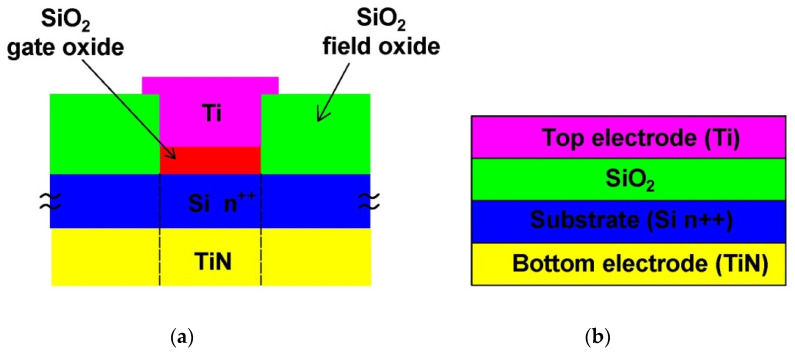
(**a**) Cross-sectional view of Ti/SiO_2_/Si(n^++^)/TiN Memristor. (**b**) Schematic of Metal-Insulator-Metal (MIM) structure.

The proposed Metal-Insulator-Metal (MIM) based Memristor model is shown in Figure 4 with the top electrode being titanium metal with a work function of 4.33 eV and bottom electrode as Titanium Nitride (TiN) with 4.2 eV metal work function. Switching between the “ON” and “OFF” states is facilitated by the active insulating (SiO_2_) between the top and bottom electrodes. The memristive devices’ switching mechanism and subsequent I-V dynamics are not disrupted by the materials used to make them.

The non-linear model implies asymmetric switching behaviour. Threshold Adaptive Memristor (TEAM) model [18] has been utilized to create memristive-type logic circuits. To improve efficiency, scientists are developing a three- and four-terminal variation. The drive current of newly developed memristors is increased since they are gated and double-gated. The complexity increases as the number of terminals increases.

Several crossbar-based logic design techniques are proposed, which allows the data process to be accomplished even without the need to transmit data from/to memory device. There are three types, namely Material Implication Logic (IMPLY), Memristor Aided Logic (MAGIC), and Memristor Ratioed Logic (MRL). For the proposed circuit implementation, we are using MRL logic as it is the only logic that is compatible with transistors [19,20,21].

### 2.3. Design and Working of 6T-CMOS SRAM

**Figure 5:**
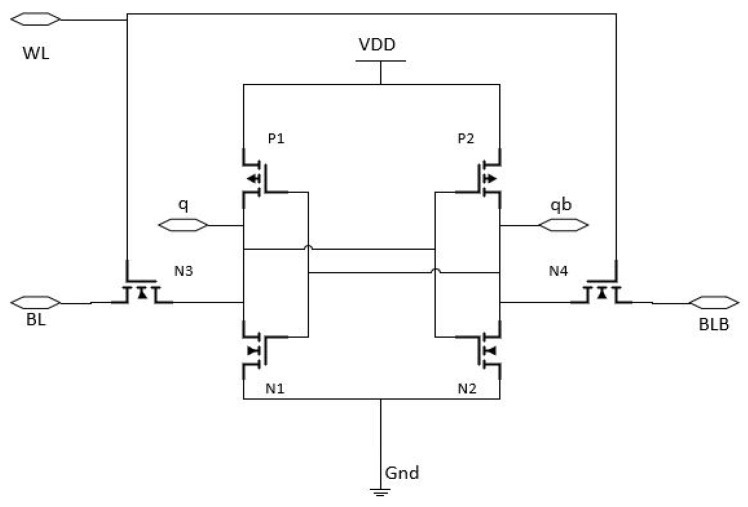
Implementation of 6T–CMOS Static RAM (SRAM) design [8].

Schematic of a 6T SRAM cell is shown in Figure 5. This design utilises two sets of inverters, P1-N1 and P2-N2, made from cross coupled CMOS transistors for reserving the bits, and two sets of access transistors, N3-N4, made from NMOS transistors for reading and writing. In order to turn on or turn off the access transistors, the word line is employed [7]. While performing a “write”, the bit lines BLB and BL are read and used as inputs. However, the bit lines carry the value that is currently read out at the time of the operation. Until power is applied, the information is kept in the SRAM memory module. 

In preparation for the “read”, both word lines and the bit lines are activated and charged. For the read mode, zero or one is stored in the SRAM cell and is recovered. The N3 and N4 access transistors are turned on after the word line is activated. The detecting amplifier picks up on the IR dip in the BL or BLB. The value of ‘q’ is calculated with the use of a sensory amplifier. To save the new value in the SRAM cell, the device must be set to “write mode” [8,9]. Changes to the SRAM cell are stored in advance when ‘q’ is set to zero or one. In standby mode, word lines are disabled by turning off access transistors N3 and N4. Any information saved in an SRAM cell will remain unchanged under these conditions.

### 2.4. FinFET Based 6T-SRAM

**Figure 6:**
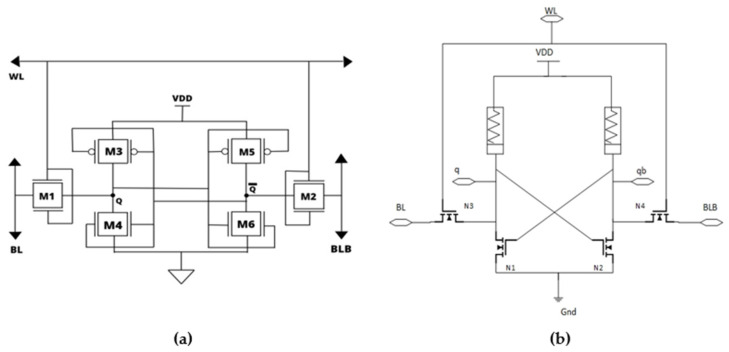
Implementation of 6T-SRAM design using (**a**) FinFET [12]. (**b**) Hybrid MOS–Memristor [22].

6T-SRAM cell with cross coupled inverters and FinFET access-transistors is shown in Figure 6a. In each of these cross coupled inverters, FinFET transistors are employed (M3, M4, M5, and M6). This architecture is similar to the CMOS-based 6T static design except for the fact that FinFET transistor is used for the implementation. FinFET-based microchips were mainstreamed in 2010 and swiftly surpassed the manufacturing nodes of 22 nm, 14 nm, and 7 nm. In compared to traditional FETs, FinFETs demonstrated decreased Short Channel Effects (SCEs), lower leakage currents, improved electrostatic stability, and optimal performance. Further, the area coverage is lesser for FinFET due to its small channel length. Hence lesser power consumption and smaller delay is achieved.

### 2.5. Hybrid MOS-Memristor Based 6T-SRAM

The hybrid combination of MOS and Memristor based 6T-SRAM circuit architecture is displayed in Figure 6b. The cell is based on a standard 6T-SRAM cell. A common variation consists of an inverter with NMOS transistors and a memristor is linked to the drains of the transistors (N1 and N2). Hence this structure is 2M-4T which will be having lesser area and power consumption [22,23,24]. M–Memristor; T–Transistor. Similar works have also been carried out for the implementation of look up tables in PROM circuits using double-gated Memristors and MOS transistors [25,26,27].

### 2.6. Basic Operations of Static RAM (SRAM)

There are three modes of operation for SRAM cells: Write, hold, and read. In Write mode, various bit values can be written to the SRAM cell to replace the original stored bit. The SRAM cell may keep data in data storage or hold mode for as long as it is plugged in [17,18]. In “Read” mode, an SRAM cell can transfer its stored data. The data is unaffected by this method of operation.

(1) Write Operation

To perform the write operation, we need to first enable the access transistors by pushing the word line high while maintaining WL = 1. Now is the time to finish the write operation. Bit Line (BL) obtains the necessary data for writing, whereas Bit Line Bar gets its complement (BLB). The cell’s status would change as a result [19,20]. When WL = 0, the word line is inactive, which means the memory retains the prior value.

(2) Hold Operation

The SRAM cell is isolated from Bit-Line (BL) and Bit-Line Bar (BLB) by FinFET access transistors if word-line (WL = 0) is not specified (M1 and M2). The latch is formed by the two cross-coupled inverters, but as long as M3 to M6 are linked to the supply voltage, they reinforce each other (VDD). Leakage current is the current flow from the VDD in this state.

(3) Read Operation

The word line is initially set high (WL = 1), which enables the latch’s access to FinFETs, allowing data to be read from the SRAM cell’s output terminal (M1 and M2). Both Bit-lines (BL and BLB) are set to ‘1′ now. Depending on the latch status, one of the bit-lines would stay pre-charged while the other would be discharged to the ground. Both bit-lines are applied to sense amplifier’s input at this point, which significantly amplify the signal before delivering the stored bit’s information.

## 3. Novel Silicon Substrate FinFET–Ti/TiN Memristor Hybrid 6T-SRAM Design

**Figure 7:**
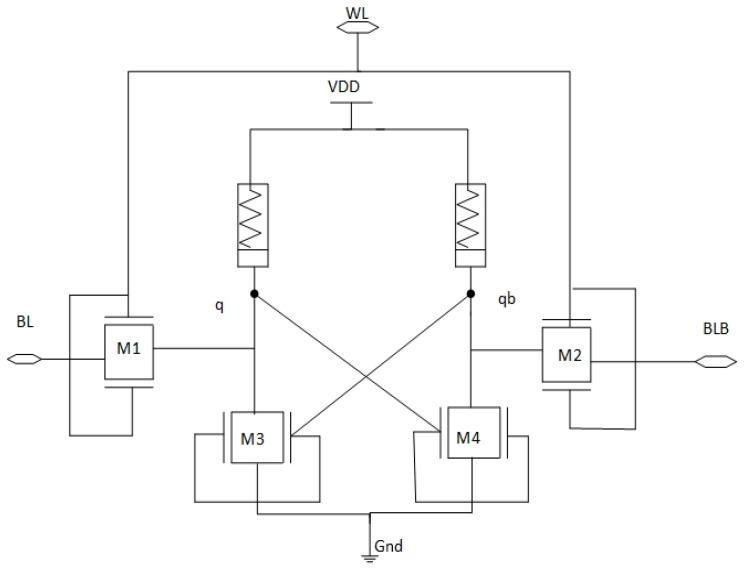
Silicon Substrate FinFET–Ti/TiN Memristor based hybrid 6T-SRAM design.

The SRAM circuit works properly with CMOS static logic, but it uses a lot of power and occupies a lot of space. The work presented in Figure 7 utilizes a hybrid silicon substrate FinFET and Ti/SiO_2_/Si(n^++^)/TiN Memristor for the implementation of 6T-SRAM circuit to achieve reduced power and area coverage. Cross coupled hybrid FINFET and memristor based inverters are used to create the memory circuit. Access transistors in static CMOS are being replaced with FinFET technology. 

The Monte Carlo (MC) approach involves the use of statistical sampling in a computer system to approximate a mathematical equation or model’s solution. It accomplishes this by feeding a model constructed with random number sequences, with the outputs serving as measures of the model’s efficacy. Monte Carlo analysis is a method for determining the probability of a particular event based on a mathematical model of a real-world system or process. In Monte Carlo, deterministic calculations are replaced by those based on a random number generator. In order to analyse and evaluate complex measurements and to experiment with the model to draw conclusions about the behaviour of the system, MC simulation is a versatile tool. Therefore, we can use this simulation to test our proposed model and draw conclusions about how it will perform in realistic settings, such as stability. In the ADE-XL setting, we use Monte-Carlo analysis to examine process and mismatch variations. Both of the delay deviations are significantly affected by the randomness of the parameters. Therefore, 2000 iterations of Monte Carlo simulations are used to assess the delay variability. 

## 4. Results & Discussion

The basic architecture for the SRAM sense amplifier using CMOS, FinFET, and Memristor are designed and implemented using the Cadence Virtuoso simulation tool. 

### 4.1. Implementation of 6T SRAM Using CMOS Technology

**Figure 8:**
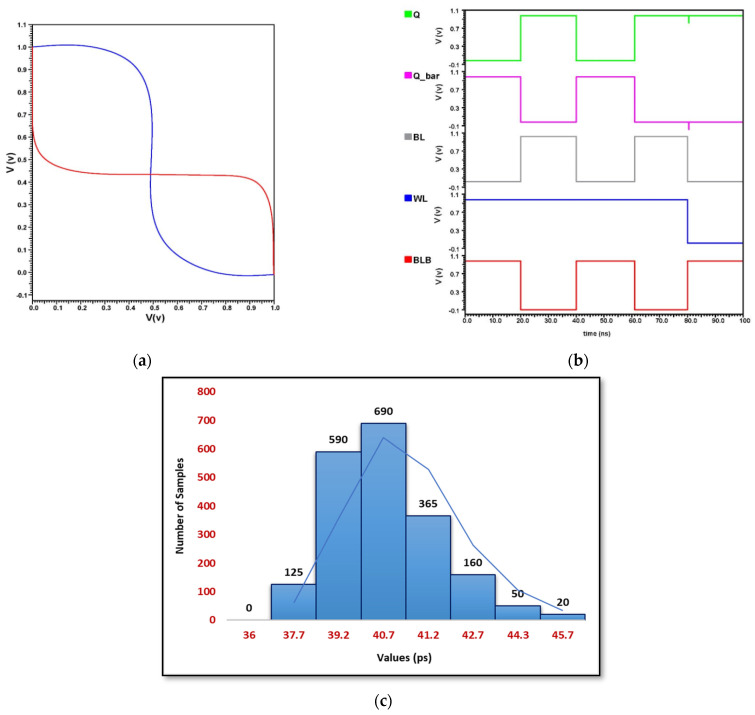
Conventional CMOS 6T-SRAM, (**a**) DC Analysis, (**b**) Transient Analysis and (**c**) Monte Carlo Simulation.

The basic 6T SRAM is implemented using Cadence Virtuoso using technology file of gpdk 45 nm, as shown in Figure 5. In the DC analysis of Figure 8a, the butterfly curve between both output nodes can be noticed. The inverter features are drawn and mirrored to create the butterfly curve, then calculating the greatest square that can be made between them. The Static Noise Margin (SNM) is determined by the length of the square’s side. The Static Noise Margin determines the SRAM’s stability. The SNM is a metric for determining how stable something is. The greater the SNM, the more stable it is. From the dc analysis, it is well observed that the stability from the 6T SRAM from static CMOS is large as SNM is large. The transient analysis in Figure 8b shows the operation of write and hold without any voltage drop in the output waveforms. This shows this circuit has excellent stability. From Figure 8c, the Monte Carlo simulation results for static CMOS SRAM have depicted an average mean value of 15.553 ps and a standard deviation of 4.8163 ps. This curve gives the value of variability of 8.45047 for the random sampling method.

### 4.2. Implementation of Proposed Ti/SiO_2_/Si(n++)/TiN Memristor 

**Figure 9:**
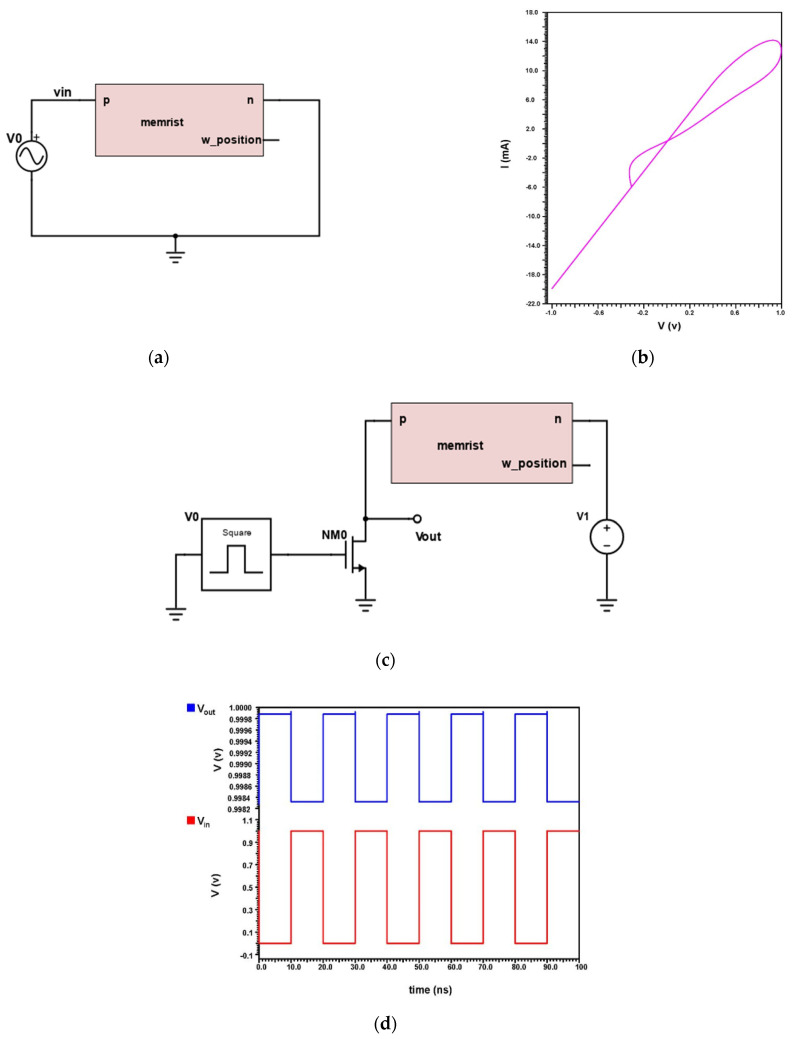
(**a**) Ti/TiN based Memristor schematic and its (**b**) I-V Characteristics, (**c**) Schematic of Memristor–MOS Transistor based Inverter and its (**d**) Transient Analysis.

The memristor is better in terms of lesser area coverage, and lesser power consumption with high speed. For determining the characteristics of the memristor, a basic circuit is implemented for determining the IV characteristics of the proposed Ti/SiO_2_/Si(n++)/TiN Memristor. The schematic of the same is depicted in Figure 9a. The IV characteristics clearly shows that the proposed device is properly working as a memristor with an evident hysteresis curve in the DC analysis graph of Figure 9b.

Figure 9c portrays a schematic view of the hybrid Memristor–MOS based inverter circuit. For the SRAM implementation, it is required to design an inverter circuit using the proposed memristor and N-channel MOS transistor. The input is given into the input of the transistor and output is from the drain end of the transistor and doped side of the memristor. The transient analysis output from the designed circuit is as expected without any drop in voltage, as plotted in Figure 9d. 

### 4.3. Implementation of Hybrid MOS-Memristor Based 6T-SRAM

**Figure 10:**
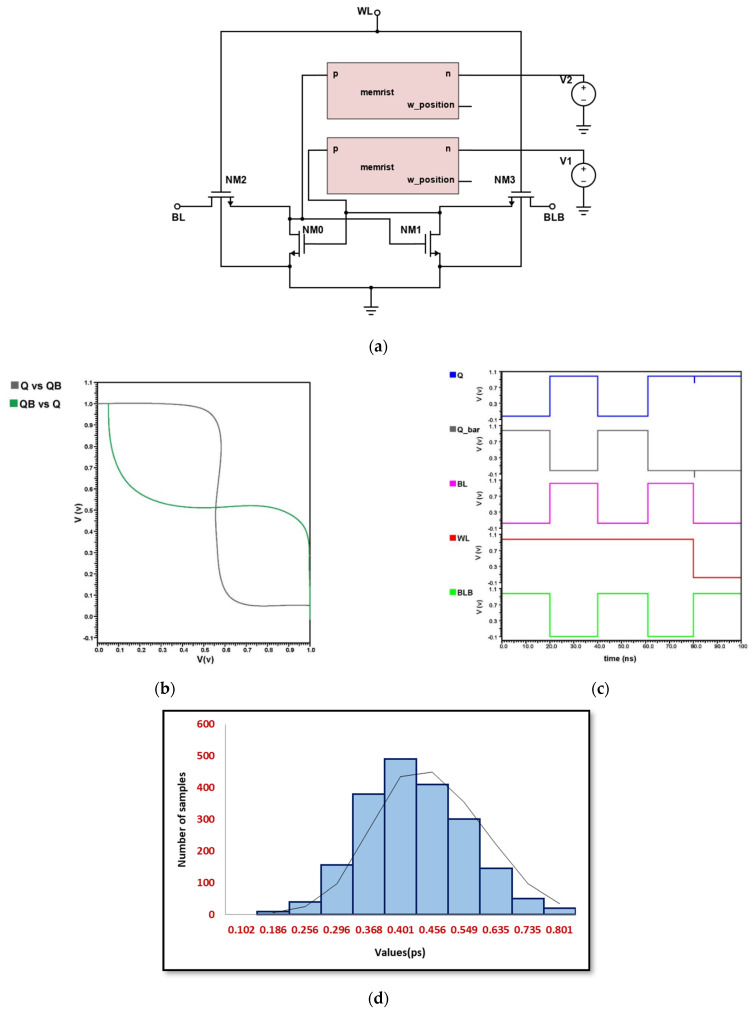
Hybrid MOS–Memristor 6T-SRAM design (**a**) Schematic, (**b**) DC Analysis, (**c**) Transient Analysis, and (**d**) Monte Carlo Simulation.

The schematic in Figure 10a consumes lesser area as two memristors are used instead of two MOS transistors for SRAM implementation. The stability of the implemented design becomes reduced, as the SNM becomes substantially lesser as compared to the static CMOS transistor based 6T SRAM. This can be seen in the subsequent analysis depicted in Figure 10b,c. We obtained a Monte Carlo curve for MOS–Memristor SRAM which has an average mean value of 0.401 ps and a standard deviation of 0.0958 ps, as shown in Figure 10d. This curve gives the value of variability of 4.18580 for the random sampling method. We obtained a reduced amount of variability when compared to the conventional model.

### 4.4. Implementation of Silicon Substrate FinFET Based 6T-SRAM

**Figure 11:**
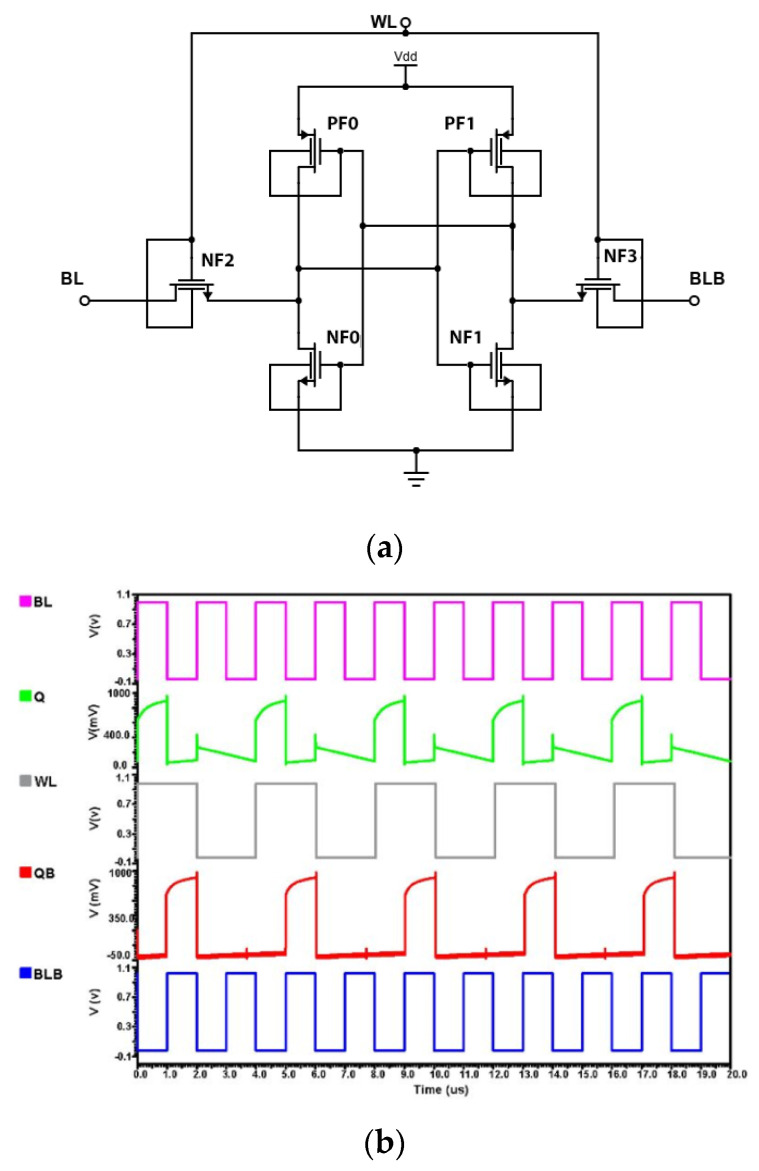
Silicon Substrate FinFET based 6T-SRAM design (**a**) Schematic and (**b**) Transient Analysis.

For smaller channel transistor designs, FinFET is more preferable over the MOS-based design. Hence, using 32 nm technology for FinFET, the design of basic 6T SRAM is implemented in Cadence Virtuoso and its transient response is analyzed. From the above analysis, it is clear that there is a voltage drop issue of FinFET because of leakage current caused due to a lesser threshold of FinFET technology. Figure 11 provides both a schematic representation of the Silicon Substrate FinFET based 6T-SRAM design and an analysis of its transient response.

### 4.5. Proposed Hybrid FinFET and Ti/SiO_2_/Si(n++)/TiN Memristor 6T-SRAM Design

**Figure 12:**
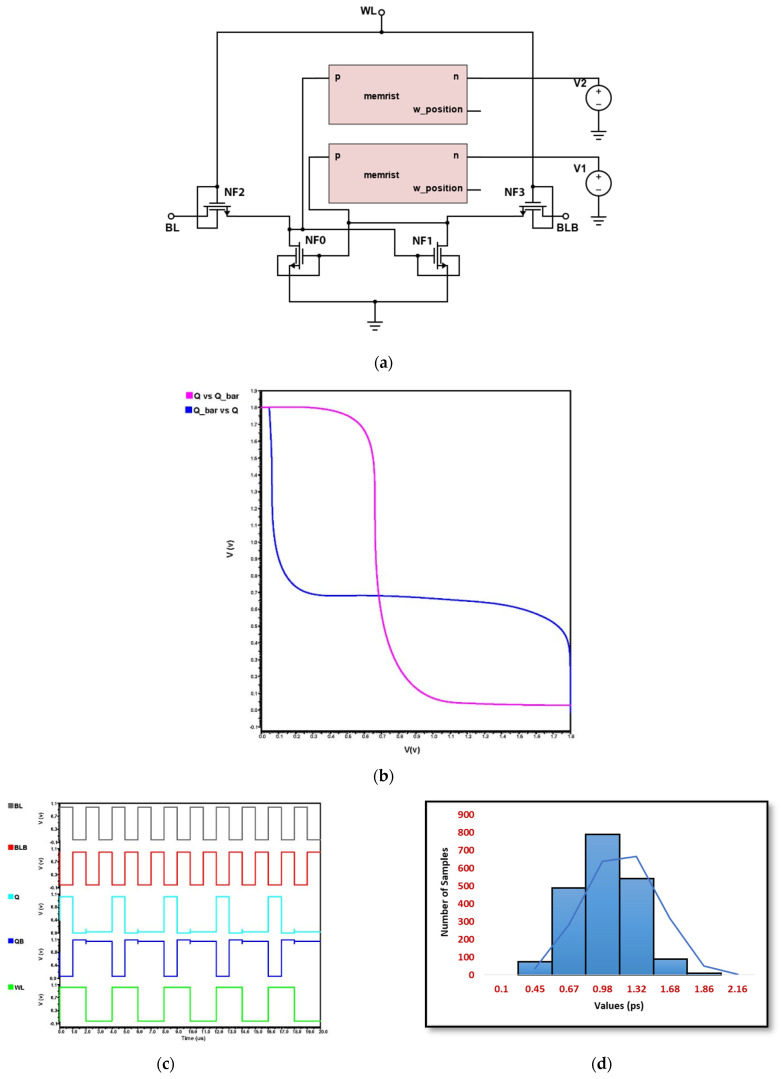
Proposed hybrid FinFET and Ti/SiO_2_/Si(n++)/TiN Memristor 6T-SRAM (**a**) Schematic, (**b**) DC Analysis, (**c**) Transient Analysis, and (**d**) Monte Carlo Simulation.

From all the above analysis carried out with the implementation of 6T-SRAM using CMOS transistors, FinFET, and hybrid MOS–Memristor, it is clear that both FinFET and hybrid MOS-Memristor design are quite stable and hence the proposed work concentrates on the 6T-SRAM design using hybrid FinFET and Ti/TiN Memristors, as implemented in Figure 12a. Memristor and FinFET are good candidates for lesser area and power consumption. In this proposed implementation, CMOS technology is replaced using FinFET technology in the hybrid CMOS–Memristor-based design. The proposed circuit and its simulation analysis are given in Figure 12a–d. From the DC analysis plot in Figure 12b, it is well understood that the SNM is quite large for the proposed hybrid design of the FinFET–Memristor which shows that the 6T-SRAM design is much more stable than the existing realization of the static memory using CMOS Transistors and Memristors. Even the voltage drop issue experienced by FinFETs during its transient analysis shown in Figure 11b is not observed in our proposed hybrid approach. 

The Monte Carlo (MC) curve for hybrid FinFET–Memristor SRAM is depicted in Figure 12d, which has an average mean value of 0.988 ps and a standard deviation of 0.153 ps. The MC analysis highlights the value with a variability of 6.4575 through the random sampling method. However, the variability of the proposed hybrid approach is lesser than static CMOS SRAM but greater than MOS–Memristor SRAM. ADE-XL utilizes Monte Carlo to investigate process and mismatch variation. Statistics affect both delay deviations. To evaluate delay variability, 2000 Monte Carlo simulations are undertaken [3]. 

**Figure 13:**
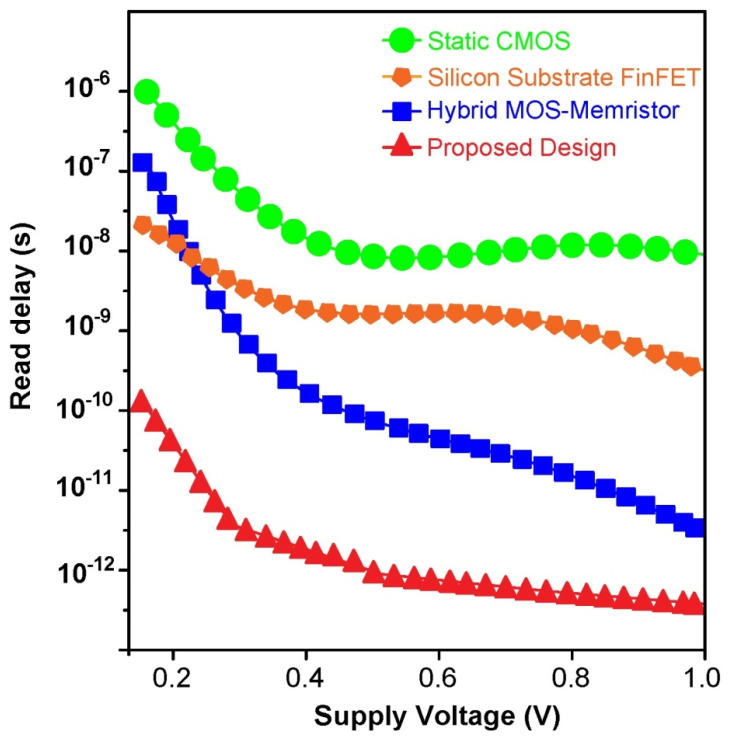
Read delay comparison of hybrid FinFET and Ti/SiO_2_/Si(n++)/TiN Memristor 6T-SRAM and traditional SRAM designs for various supply voltages.

During pre-charging to VDD, the Bit Lines are contended high for the read operation. While the pull down fin transistor is high, the bit at the node output of “Inverter 1” implemented using MIM Memristor will become “0”. Again, when the BL releases from VDD, the differential bit line sense amplifier triggers. Read delay is a parameter used to quantify SRAM cell read performance. The read-delay is the time between 50% word line (WL) excitation and 10% bit line pre-charged voltage variance. The read delay readings of the proposed hybrid FinFET and Ti/SiO_2_/Si(n++)/TiN Memristor based 6T SRAM is compared to traditional SRAM designs for various supply voltages in Figure 13. The comparative results reveal that the proposed 6T SRAM with hybrid design of sense amplifier outstands the conventional designs.

**Figure 14:**
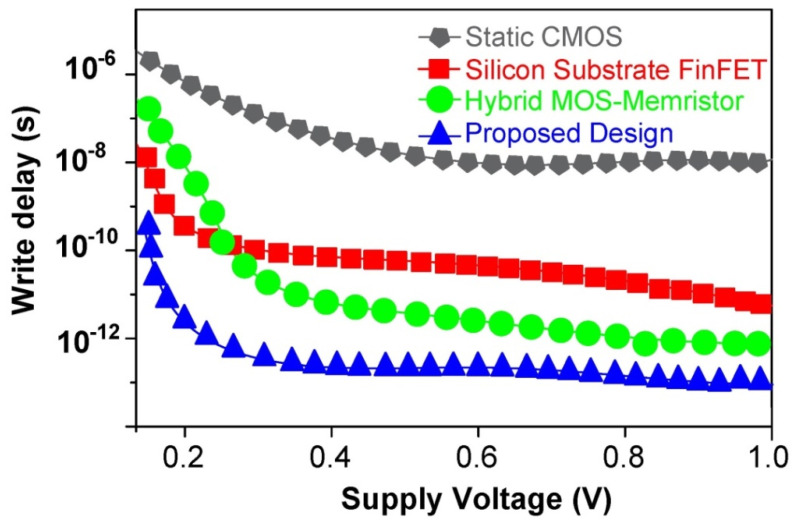
Write delay comparison of hybrid FinFET and Ti/SiO_2_/Si(n++)/TiN Memristor 6T-SRAM and traditional SRAM designs for various supply voltages.

Hybrid FinFET-MIM Memristor-based static memory cells can also be configured to write. Two FinFETs NF2 and NF3, depending on the memory cell bit to be written, can accomplish this. Charging BL to VDD will write bit “1” into the cell. By asserting NF2’s gate control input (WL) high, this is achieved. With source input BL high, FinFET (NF2) output (Q) settles to “1” and QB to “0”. The simultaneous assertion of write enable line and high WL triggers this. Deactivating the BLB line to 0 V writes “0” into the intended SRAM cell. This, in turn, activates WL to break the coupling between the two Memristor inverters, causing the node Q to store “0” with the assistance of the second access FinFET (NF3). From Figure 14, the suggested structure with FinFET and Ti/SiO_2_/Si(n++)/TiN Memristor has a low write delay compared to other SRAM designs reported in the literature.

**Figure 15:**
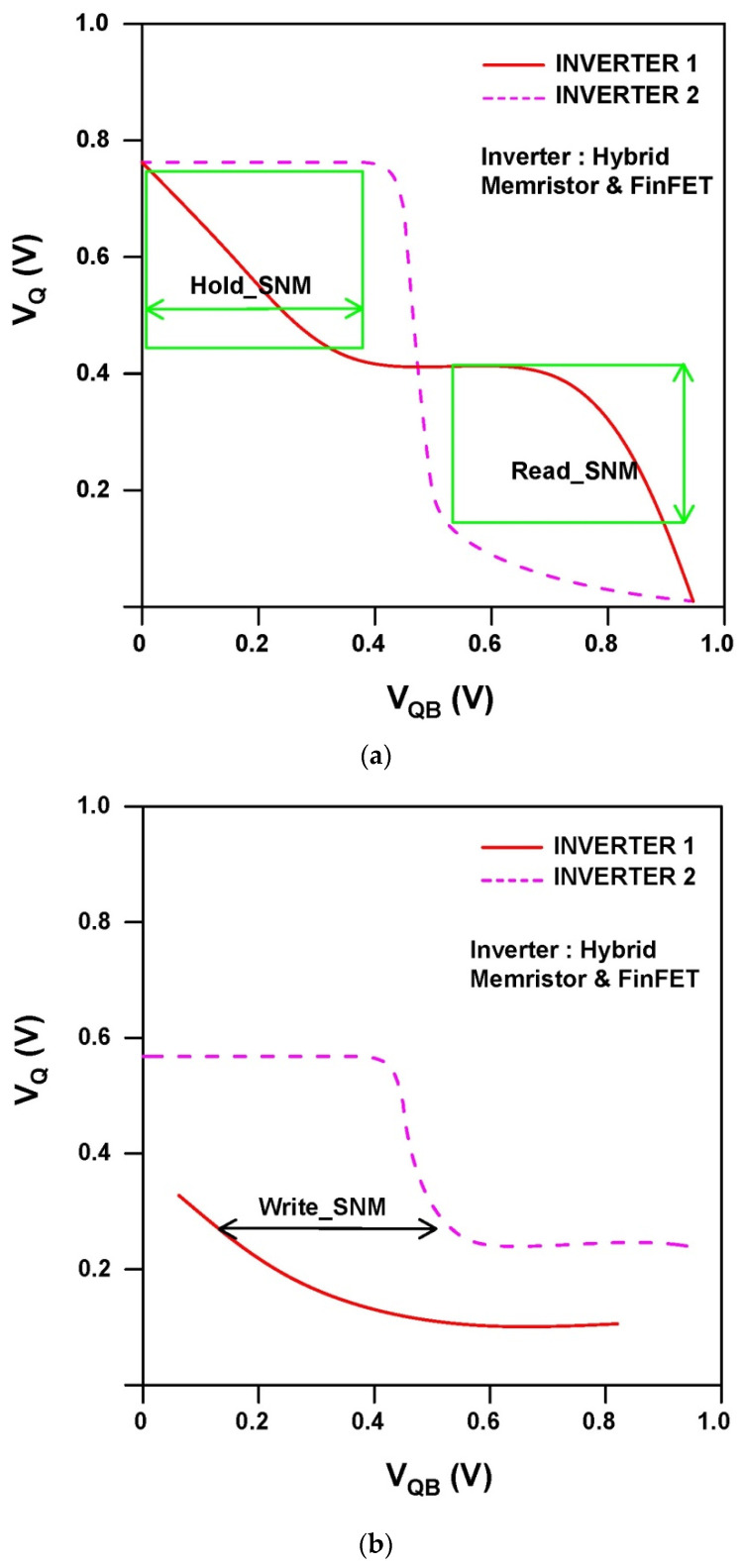
Proposed hybrid FinFET and Ti/SiO_2_/Si(n++)/TiN Memristor 6T-SRAM. (**a**) Hold and Read Static Noise Margin (SNM). (**b**) Write SNM.

**Figure 16:**
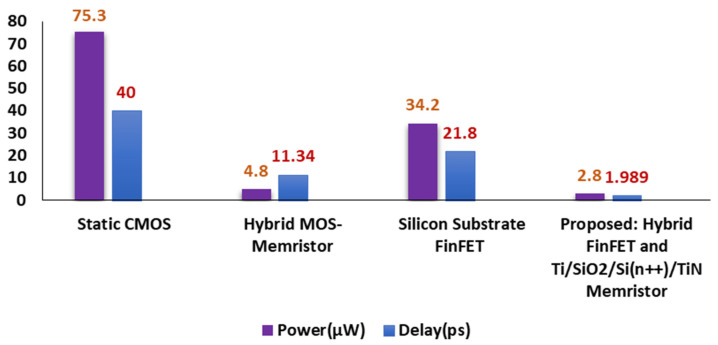
Power and Delay comparison of various 6T-SRAM designs with the proposed hybrid Si substrate FinFET–Ti/TiN Memristor.

Hold, read and write Static Noise Margin (SNM) measurements for the proposed sense amplifier design of static RAM are displayed in Figure 15a,b. The findings demonstrate that the suggested device has high noise endurance, as the SNM window frames for read and write operations are sufficiently accurate. Table 1 and Figure 16 show how the suggested designs stack up against the current domino logic topologies in terms of power and efficiency, respectively. Using fewer transistors and activating them only when necessary contributes to the significant drop in power requirements. This exemplifies the swift operation of the proposed hybrid FinFET and Ti/SiO_2_/Si(n++)/TiN Memristor. The reported design has a static and dynamic power dissipation of 6.59 nW and 2.71 µW to deliver an average power dissipation of 2.8 µW. 

## 5. Conclusions

This work proposes new circuits and expands on others already in use to largely address the performance issues in FinFET devices. With different configurations for SRAM using different technologies, such as static CMOS (45 nm technology), FinFET (32 nm technology) and Memristor for implementing the 6T-SRAM design in Cadence Virtuoso. On repeated analysis for each of the existing technologies, we conceived of a novel hybrid design of a Silicon (Si) substrate FinFET and Ti/SiO_2_/Si(n++)/TiN Memristor based SRAM. The design is compared with conventional CMOS 6T SRAM, Hybrid CMOS- memristor, and FinFET based 6T SRAM. The reported design is consuming less area as compared to others. Further comparison is done with respect to power and delay. It is much more evident that the proposed hybrid FinFET-Memristor is consuming less power and has minimal delay. Further, from the DC analysis it is still clear that the Static Noise Margin (SNM) is more for hybrid FinFET–Memristor when compared to hybrid CMOS–Memristor design. The Monte-Carlo analysis performed has also substantiated the stability of the proposed device and the results prove that the suggested design variability is relatively higher than that of the hybrid CMOS memristor design but is still superior to static CMOS SRAM.

## Figures and Tables

**Table 1:** Comparison of Power, Delay and Area for various SRAM implementations.

6T-SRAM Design	Technology	Power (µW)	Delay (ps)	No. of Devices
Static CMOS [8]	45 nm	75.3	40	6T
Hybrid MOS-Memristor [22]	45 nm	4.8	11.34	4T + 2M
Silicon Substrate FinFET [12]	32 nm	34.2	21.8	6FT
**Proposed:** **Hybrid FinFET and Ti/SiO_2_/Si(n++)/TiN Memristor**	32 nm	2.8	1.989	4FT + 2M

T–MOS Transistor (45 nm); M–Memristor (3 nm); FT–FinFET (32 nm).

## Data Availability

Not applicable.
